# Availability of emergency obstetric care (EmOC) among public and private health facilities in rural northwest Bangladesh

**DOI:** 10.1186/s12889-015-1405-2

**Published:** 2015-01-31

**Authors:** Shegufta S Sikder, Alain B Labrique, Hasmot Ali, Abu AM Hanif, Rolf DW Klemm, Sucheta Mehra, Keith P West, Parul Christian

**Affiliations:** Department of International Health, Johns Hopkins Bloomberg School of Public Health, Baltimore, MD USA; The JiVitA Maternal and Child Health Research Project, Gaibandha, Bangladesh

**Keywords:** Maternal health, Rural Bangladesh, Emergency obstetric care, Quality of care, Global health, Human resources for health

## Abstract

**Background:**

Although safe motherhood strategies recommend that women seek timely care from health facilities for obstetric complications, few studies have described facility availability of emergency obstetric care (EmOC). We sought to describe and compare availability and readiness to provide EmOC among public and private health facilities commonly visited for pregnancy-related complications in two districts of northwest Bangladesh. We also described aspects of financial and geographic access to healthcare and key constraints to EmOC provision.

**Methods:**

Using data from a large population-based community trial, we identified and surveyed the 14 health facilities (7 public, 7 private) most frequently visited for obstetric complications and near misses as reported by women. Availability of EmOC was based on provision of medical services, assessed through clinician interviews and record review. Levels of EmOC availability were defined as basic or comprehensive. Readiness for EmOC provision was based on scores in four categories: staffing, equipment, laboratory capacity, and medicines. Readiness scores were calculated using unweighted averages. Costs of C-section procedures and geographic locations of facilities were described. Textual analysis was used to identify key constraints.

**Results:**

The seven surveyed private facilities offered comprehensive EmOC compared to four of the seven public facilities. With 100% representing full readiness, mean EmOC readiness was 81% (range: 63%-91%) among surveyed private facilities compared to 67% (range: 48%-91%) in public facilities (p = 0.040). Surveyed public clinics had low scores on staffing and laboratory capacity (69%; 50%). The mean cost of the C-section procedure in private clinics was $77 (standard deviation: $16) and free in public facilities. The public sub-district facilities were the only facilities located in rural areas, with none providing comprehensive EmOC. Shortages in specialized staff were listed as the main barrier to EmOC provision in public facilities.

**Conclusions:**

Although EmOC availability and readiness was higher among the surveyed seven most commonly visited private clinics, public facilities appeared to be more affordable for C-section and more geographically accessible. Strategies to retain anesthesiologists and surgeons, such as non-financial incentives, are needed to improve EmOC provision in the public sector. Centralized blood banks are recommended to streamline safe blood acquisition for obstetric surgeries.

## Background

Improving provision of emergency obstetric care remains the cornerstone of Bangladesh’s maternal health strategy as well as global safe motherhood strategies [1,2]. Although most obstetric complications (defined as acute conditions such as postpartum hemorrhage, sepsis, eclampsia, and obstructed labor that can cause maternal death [3]) cannot be predicted, the majority can be treated with timely provision of a package of evidence-based interventions known as emergency obstetric care (EmOC) [4,5]. The availability of EmOC is considered to be an indicator of how well a health system is prepared to manage conditions leading to acute maternal morbidity and mortality [6,7].

The World Health Organization (WHO), in its *2010 Monitoring Indicators for Health Systems Handbook*, defines availability as the “physical provision of health services” and readiness as “capacity to deliver health services” [5]. Studies on availability of emergency obstetric care have traditionally distinguished between comprehensive EmOC (surgical services of C-section and blood transfusion in addition to basic obstetric services) and basic EmOC (non-surgical obstetric interventions). According to the 2012 *Health Bulletin* produced by Bangladesh’s Ministry of Health and Family Welfare, all 26 tertiary medical college hospitals, 59 district hospitals, 132 sub-district health complexes, and 63 maternal and family welfare centers are recorded to provide comprehensive emergency obstetric care [8]. Research indicates, however, that these records may over-estimate actual availability of services. For example, although districts hospitals in Sylhet and Khulna districts of Bangladesh were categorized as providing comprehensive emergency obstetric care, studies in these districts suggested sub-optimal availability of emergency obstetric care among surveyed public facilities, particularly due to staffing constraints [9,10].

EmOC assessments in Bangladesh have been largely limited to public facilities without inclusion of private facilities, which are documented to account for a growing proportion of institutional deliveries in Bangladesh [9,11]. Recent estimates suggest that 15% of facility-based births nationwide occur in the private sector, compared to 12% in the public sector [12]. Although the number of private clinics in rural areas has tripled over the past decade [8], little data exists comparing the availability of EmOC services in the public to the private sector. National reviews of maternity service provision in Bangladesh recommend focusing service improvement on high-volume health facilities that capture the majority of women seeking care for complications of pregnancy and childbirth [9,11]. Recognizing that national health budgets are limited and regional/district resources are scarce, Travis and colleagues suggest focusing on improvements at health facilities that capture a large proportion of women of reproductive age who seek care for obstetric complications [6,11].

In alignment with this service delivery improvement perspective, this analysis sought to explore the availability and readiness for emergency obstetric care provision among commonly visited health facilities (public and private) for pregnancy-related health care in two districts of rural northwest Bangladesh. The goal of this analysis is to inform recommendations to improve the provision of emergency obstetric care at the high-volume facilities where most women access services. We compared EmOC availability and readiness between public and private facilities surveyed in this study. In addition, we described aspects of geographic and financial access to services among these facilities and key constraints to EmOC provision in order to inform service improvement recommendations. These data may be informative for district planning to improve health service delivery for emergency obstetric care. In addition, these recommendations may inform Bangladesh’s maternal health strategy and safe motherhood strategies, which are focused on reducing maternal deaths through improved utilization and access to emergency obstetric care.

## Methods

### Sampling of health facilities

We conducted assessments of health facilities in Gaibandha and Rangpur Districts in Rangpur Division [13]. This area was selected since it represented typical rural populations in Bangladesh based on population density (~1000 people per square kilometer), rural, agrarian characteristics (villages surrounded by rice fields, linked by unpaved roads), and economic and public health indicators [13,14]. With a population of 2.3 million people, Gaibandha District in northwest Bangladesh includes 14 private clinics and four public hospitals (as of December 2011), with the majority of facilities located in Gaibandha Town (*Sadar*). Bordering Gaibandha District to the northeast is Rangpur District, which serves as a commercial hub in northern Bangladesh with a population of 2.8 million [14]. The town of Rangpur (Rangpur *Sadar*) includes a medical college hospital, which serves as a tertiary facility, a public hospital, and more than 99 private clinics (as of December 2011). While this study included all public health facilities in the study area, we were unable to survey all 113 private facilities in the study area. (The large number of private facilities in these two districts is indicative of the proliferation of private facilities across the country [8].) In alignment with the service delivery improvement perspective of this analysis, we focused on surveying high-volume private clinics for pregnancy-related complications in these two districts. As literature indicates that facility use is influenced by the quality and level of care provided [3,9], these selected private facilities may have higher service quality compared to the private clinics that were not surveyed in this study. While the surveyed private clinics may not be representative of all private clinics, they represent the high-volume health facilities that capture the majority of women who seek private facilities for obstetric complications. For districts with limited health budgets, focusing on service improvement at this selection of private clinics is likely to reach a large proportion of women of reproductive age with pregnancy-related complications.

**Figure 1:**
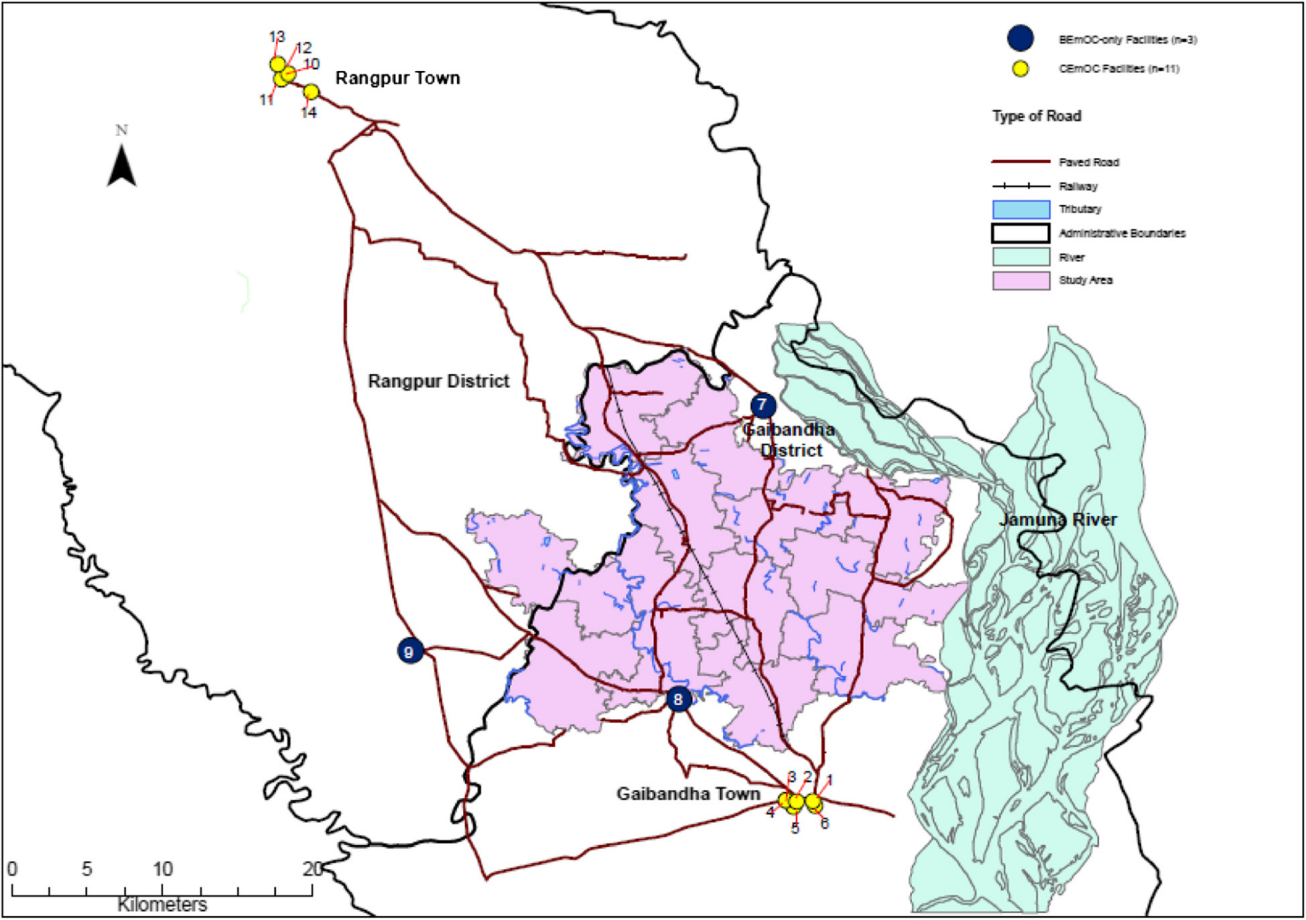
**Location of 14 health facilities surveyed in Gaibandha and Rangpur Districts, by level of care provided.** The JiVitA study area is shown in pink, while black lines denote administrative boundaries between districts and paved roads. All facilities that offer comprehensive emergency obstetric care (shown as yellow circles; numbers 1–6 and 10–14) are located in the main towns. The three rural sub-district public facilities (shown as blue circles; numbers 7–9) offer basic emergency obstetric care only.

Rangpur District included one rural hospital outside of the main town. By the national highway connecting the two towns, the distance between Rangpur Town and Gaibandha Town is approximately 65 kilometers. Figure 1 illustrates the location of the 14 health facilities included in our survey. All facilities providing comprehensive EmOC (shown as yellow circles) were located in the main towns, with the three public facilities in the rural areas providing BEmOC only (shown as blue circles). By motorized transport, travel time between the center of the study area to CEmOC facilities in the main towns is between one to two hours.

We focused on availability of obstetric services at health facilities since current WHO guidelines recommend that emergency obstetric care be administered in facility settings [15,16]. For study participants enrolled in the parent JiVitA-3 study, we used data on reported care seeking for self-reported symptoms of obstetric complications or near misses (as defined elsewhere [17]) to identify the most frequently visited health facilities. Conducted in Gaibandha and Rangpur between 2007 and 2011, the JiVitA-3 community-randomized controlled trial enrolled 44,567 pregnant women to assess the effect of daily antenatal supplementation with multiple micronutrients, compared to iron-folic acid, on six-month infant mortality (Clintrials.gov #NCT00860470) [18]. Women were eligible for the parent trial if they could become pregnant (were of reproductive age 13–45 years, married and living with their husbands, not sterilized or menopausal, and whose husbands were not sterilized) [13]. Eligible women were visited by field workers every five weeks to administer pregnancy tests for missed menses. Women who were identified as pregnant, based on urine tests, were asked for consent to enroll in the parent trial. If they consented, women were interviewed upon enrollment and throughout the pregnancy and postpartum period on morbidity symptoms, care seeking, and assessments of nutritional status, among other data.

One month following the end of their pregnancies, women were asked whether they experienced a series of morbidities in the 30 days before, during, or after their pregnancies had ended. Women who reported experiencing any morbidity for longer than one day were asked about the health provider or facility from which they sought care. Those who said that they felt that they nearly died at any time during pregnancy, delivery, or 30 days postpartum were asked about the symptoms they experienced and up to four health providers from whom they sought care. During these interviews, both the type of provider or health facility as well as the identification number of the provider was recorded. The study area was mapped using a Geographic Information System (GIS). This mapping allowed for identification of the locations of the health providers or facilities.

Using these available data, we calculated the total number of visits women made to health facilities between December 2007 and June 2011 for symptoms comprising obstetric complications and near misses. Out of the 42,796 women with pregnancy outcomes recorded between December 2007 and June 2011, 27% (n = 11,384) reported symptoms of obstetric complications or near misses, with 79% (n = 8,326) seeking care for these conditions. Table 1 shows the volume of total visits at each health facility made for reported obstetric complications or near misses. Private-sector facilities accounted for 46% of total visits made to health facilities, while public-sector facilities accounted for 54% of visits.

**Table 1:** **Health facilities visited by women reporting obstetric complications and near misses in the JiVitA-3 trial**

**Health facility**	**% of total visits to health facilities (# of total visits to each facility/ total # of visits to health facilities)**
***Public facilities***	
Maternal and Child Welfare Center, Gaibandha	16% (465)
District (*Sadar*) Hospital, Gaibandha	10% (291)
Sundorgonj Sub-district (*Upazila*) Health Complex	9% (262)
Medical College Hospital, Rangpur	8% (233)
Sadallapur Sub-district *(Upazila)* Health Complex	5% (145)
Maternal and Child Welfare Center, Rangpur	4% (116)
Pirgonj Sub-district *(Upazila)* Health Complex	1% (29)
Other (Individual providers)	2% (58)
**Total – public facilities**	**54% (1,571/2,909)**
***Private clinics***	
Rabeya Clinic, Gaibandha	8% (233)
Gaibandha Clinic, Gaibandha	6% (178)
Update Clinic, Rangpur	6% (174)
Ideal Clinic, Rangpur	5% (146)
Islami Bank Community Hospital, Rangpur	5% (144)
Oishi Clinic, Gaibandha	5% (143)
Pulse Clinic, Gaibandha	3% (87)
Other (Individual clinics)	8% (233)
**Total – private clinics**	**46% (1,338/2,909)**

Table 1 illustrates the 14 most frequently visited health facilities among women reporting obstetric complications and near misses in the JiVitA-3 trial.

For facility assessments, we selected the 14 most frequently visited health facilities, including all seven public facilities located in the two districts and the seven most frequently visited private facilities. Collectively, the 14 selected facilities represented 90% of the total visits made to health facilities for obstetric complications reported by study participants (Table 1). We purposively selected the most frequently visited private clinics to characterize EmOC readiness at high-volume facilities for pregnancy-related complications (Table 1). The seven surveyed private clinics accounted for 83% of the visits made to private clinics by study participants for reported pregnancy-related complications. Recognizing that the selected private facilities may not be representative of all private facilities in the study area, we selected the seven most high-volume private facilities in order to inform service delivery improvement recommendations that affect the majority of women seeking obstetric care. Individual formal providers who provided care outside of health facilities were excluded since WHO guidelines recommend that emergency obstetric care be administered in health facility settings [15,16]. Informal providers were excluded from this analysis since they are not authorized to provide EmOC services in Bangladesh [8].

### Interview protocols

To assess emergency obstetric care availability and readiness at surveyed health facilities, we adapted tools developed by the Averting Maternal Death and Disability (AMDD) group at Columbia University [19] for the context of Bangladesh to account for providers who work in multiple sectors and availability of medicines at pharmacy shops adjacent to health facilities.

The interview team consisted of the first author (SSS, doctoral graduate with specialization in maternal health and disease epidemiology and control) and a nurse-midwife (with a three-year training in nursing, a 12-month course in standard midwifery, and a six-week training in emergency obstetric care). Modules were administered in Bengali and both team members took detailed notes, as these interviews were not tape-recorded. The interview team compared and harmonized notes within 24 hours following the interview to discuss and resolve any discrepancies.

To assess readiness for EmOC provision, we administered standardized modules to health administrators and to clinicians. Interviews with administrators included modules on staffing, referral capacity, service hours, costs of services, and infrastructure as well as a walk-through of the facility to assess presence and functionality of key equipment and medicines required for EmOC. Clinicians were interviewed about provision of services for EmOC and use of evidence-based practices. We supplemented clinician interviews with review of maternity ward registers, delivery registers, and general admissions registers for records of service provision in the three months preceding surveys. Both sets of respondents were asked to describe constraints to EmOC provision and potential solutions. These facility surveys were conducted between October 2011 and January 2012.

To further assess availability of essential medicines for EmOC, safe blood acquisition, and referral, we also interviewed owners of the two pharmacy shops located closest to each health facility, the deputy director of the largest NGO in the two districts responsible for safe blood distribution (called *Sandhani*), and program managers at an NGO named BRAC that provided referral linkages from rural communities to health facilities. We interviewed two respondents from the Ministry of Health and Family Welfare (MOHFW) responsible for personnel policies on constraints to service provision.

The Institutional Review Board at the Johns Hopkins Bloomberg School of Public Health determined this study to be non-human subjects research (IRB 00000287). The parent trial, JiVitA-3, from which the surveyed health facilities were identified, received IRB approval from the Johns Hopkins Bloomberg School of Public Health (IRB 00000570) and the Bangladesh Medical Research Council (BMRC Reference Number 458).

### Analytic definitions and data analysis

The definitions of EmOC availability and readiness used for this study were based on the 2010 Service Availability and Readiness Assessment methodology developed by WHO [5]. We used the term “EmOC availability” to refer to the physical provision of medical services, known as signal functions, over the past three months. Two main levels of care were defined: basic emergency obstetric care (BEmOC) and comprehensive emergency obstetric care (CEmOC), with detail on classifications shown in Table 2. These EmOC levels were further adapted into two categories: 1) Facilities offering CEmOC, which included facilities offering all nine services as well as those offering all comprehensive services except for one to two of the basic functions, and 2) BEmOC-only facilities, which included facilities offering all seven basic signal functions as well as those offering all basic functions minus one or two services [4,5,20].

**Table 2:** **Classification of emergency obstetric care by availability of medical services, or signal functions** [4,5,19]

**Level of emergency obstetric care**	**Provision of signal functions**
**Basic emergency obstetric care (BEmOC)**	1. Administration of parenteral antibiotics (for postpartum sepsis or complications of abortion)
	2. Administration of uterotonic drugs (i.e. parenteral oxytocin) (for prolonged or obstructed labor or postpartum hemorrhage)
	3. Administration of parenteral anticonvulsants (i.e. magnesium sulfate) (for pre-eclampsia and eclampsia)
	4. Manual removal of placenta (for postpartum hemorrhage)
	5. Removal of retained products (e.g. manual vacuum extraction, dilation and curettage) (for postpartum sepsis, postpartum hemorrhage, or complications of abortion)
	6. Assisted vaginal delivery (e.g. vacuum extraction, forceps delivery) (for prolonged or obstructed labor)
	7. Basic neonatal resuscitation (e.g. with bag and mask) (for prolonged or obstructed labor, pre-eclampsia or eclampsia, or newborn distress)
**Comprehensive emergency obstetric care (CEmOC)**	CEmOC facilities offer all seven basic EmOC signal functions as well as:
	8. Surgery (e.g. Caesarean section) (for antepartum hemorrhage, postpartum hemorrhage, pre-eclampsia or eclampsia, prolonged or obstructed labor, postpartum sepsis, complications of abortion, newborn distress, uterine rupture, or ectopic pregnancy)
	9. Blood transfusion (for antepartum hemorrhage, postpartum hemorrhage, complications of abortion, ectopic pregnancy, uterine rupture)

Facilities that offer the seven basic signal functions are considered BEmOC facilities. Facilities that offer all seven basic signal functions as well as the two comprehensive medical services are considered CEmOC facilities [4,5,19].

“EmOC readiness” referred to the capacity of health facilities to provide signal functions as measured through four categories, including: 1) trained staff and guidelines, 2) equipment and supplies, 3) laboratory capacity, and 4) medicines and commodities. The criteria for each category, based on the 2010 *WHO Monitoring Indicators for Health Systems Handbook*, are presented in Table 3. We used the terms “private” and “public” to refer to the sector in which health care was provided. The public sector included health care facilities established by the government, while the private sector included for-profit clinics.

**Table 3:** **Criteria for categories comprising emergency obstetric care readiness scores, by level of services provided**

**Categories**	**Basic Emergency Obstetric Care (BEmOC)**	**Comprehensive Emergency Obstetric Care (CEmOC)**
Staffing guidelines and training	- Guidelines for Integrated Management of Pregnancy and Childbirth (IMPAC)	- BEmOC Requirements +
		- Guidelines for CEmOC +
	- Staff trained in IMPAC	- Staff trained in CEmOC +
		- Surgeon and anesthesiologist on staff 24/7
Equipment/supplies	- Emergency transport	- BEmOC Requirements +
	- Examination light	- Anesthesia equipment +
	- Suction apparatus	- External heat source
	- Manual vacuum extractor	
	- Vacuum aspirator or dilatation & curettage kit	
	- Newborn bag and mask (Ambubag)	
Laboratory capacity	N/A	Cross-matching capacity
Medicines and commodities	- Partograph	- BEmOC Requirements +
	- Gloves	- No Shortage of blood in prior 3 months +
	- Injectable uterotonic	- Blood obtained only from national or regional blood bank OR Blood obtained from other sources but screened for HIV and other transfusion-transmissible infections
	- Injectable antibiotic	
	- Magnesium sulfate	
	- IV solution with infusion set	
**EmOC readiness score**	**Unweighted average of percentages in each category**	

Criteria for the four readiness categories are based on the 2010 *WHO Monitoring Indicators for Health Systems Handbook* [5].

Facility readiness for EmOC provision was expressed as a mean percentage and calculated as the unweighted average of percentages in the four categories to represent the cumulative availability of components required to provide EmOC. Within each category, readiness scores were calculated as the percentage of items present and functioning on the day of survey out of the total number of items in that category (criteria shown in Table 3). For example, a surveyed facility that had all the required medicines and commodities in its category would receive a readiness score of 100% for medicines and commodities. For each facility, overall readiness was calculated as the mean percentage of scores in the four categories. These overall scores were averaged to obtain a mean readiness score for surveyed public and private facilities. Using Stata 11 [21], Mann–Whitney tests were used to calculate p-values for EmOC availability and readiness among facilities in the public sector compared to facilities in the private sector.

The open-ended interview sections were analyzed using textual analysis. Using Atlas.ti [22], the text was coded line by line to identify key themes regarding constraints to EmOC provision and potential solutions. We also included descriptive data on financial costs of C-sections at facilities offering obstetric surgery. ArcGIS software [23] was used to create maps showing locations of health facilities relative to the study area.

## Results

### EmOC availability and readiness: summary of results

Table 4 presents general characteristics of the surveyed facilities (n = 14). Two of the public facilities (the Maternal and Child Welfare Clinic in Gaibandha and Sadallapur Sub-district Health Complex) were established before Bangladesh’s independence in 1971, while the oldest private clinic in our sample (Gaibandha Clinic) was established in 1991. In the public sector, bed capacity ranged from the 20-bed Maternal and Child Welfare Clinic (MCWC) in Gaibandha to the 600-bed Medical College Hospital in Rangpur. Bed capacity in the private sector ranged from 10 to 30 beds, though five of the surveyed clinics were in the process of expanding. Surveyed public facilities handled a total of 10,377 deliveries between January 2011 and October 2011, compared to 3,217 deliveries in surveyed private clinics (Table 4).

**Table 4:** **Characteristics of 14 health facilities surveyed between October 2011-January 2012, ordered by number of beds**

**Health service provider**	**Number of beds**	**Year of establishment**	**Health staff who attend deliveries (doctors, nurses, nurse-midwives, and family welfare visitors** ^**c**^ **)**	**Volume of deliveries from Jan 2011-Oct 2012**
***Public facilities***				
Maternal and Child Welfare Center, Gaibandha	20	1940	2 doctors, 7 FWVs, 1 nurse-midwife, 1 *dai* nurse	2,630
Sundorgonj Sub-district (*Upazila*) Health Complex	31 (50)	1983	4 doctors, 8 nurses	270
Sadallapur Sub-district *(Upazila)* Health Complex	31 (50)	1968	3 doctors, 3 FWVs, 5 nurses	111
Pirgonj Sub-district *(Upazila)* Health Complex	50	1975	8 doctors, 14 nurses	695
District (*Sadar*) Hospital, Gaibandha	100	1980	4 doctors, 29 nurses	590
Maternal and Child Welfare Center, Rangpur	100	1976	2 full time-doctors (1 pair), 6 FWVs, 1 *dai* nurse, 1 nurse	1,054
Medical College Hospital, Rangpur	600 (1,000)	1976	200 doctors, 298 nurses	5,027
**Total**				**10,377**
***Private facilities***				
Private Clinic #1, Gaibandha	10 (15)	2001	1 full-time doctor, 6 on-call doctors, 2 diploma nurses, 5 non-diploma nurses	390
Private Clinic #2, Gaibandha	10 (15)	1999	1 full-time doctor, 7 on-call doctors, 1 diploma nurse, 4 non-diploma nurses	196
Private Clinic #3, Gaibandha	10 (22)	1995	3 full-time doctors, 5 on-call doctors, 3 diploma nurses	737
Private Clinic #4, Gaibandha	20 (100)	1991	3 full-time doctors, 3 on-call doctors, 8 non-diploma nurses	704
Private Clinic #1, Rangpur	24	1996	6 full-time doctors, 70 on-call doctors, 21 non-diploma nurses	500
Private Clinic #2, Rangpur	27 (100)	1996	3 full-time doctors, 14 on-call doctors, 2 diploma nurses, 7 non-diploma nurses	274
Islami Bank Community Hospital, Rangpur	30	2001	16 full-time doctors, 6 on-call doctors, 10 diploma nurses, 13 non-diploma nurses	416
**Total**	**-**	**-**	**-**	**3,217**

These data was gathered from facility records and facility surveys conducted with health administrators. The number of beds refer to current bed capacity, while any numbers in parentheses indicate planned upgrades. Family welfare visitors (FWVs) are trained to perform normal deliveries and are considered within the definition of skilled births attendants used in the Bangladesh Demographic and Health Surveys [12].

Of the 14 surveyed facilities, 79% (n = 11) offered CEmOC, while 21% (n = 3) offered BEmOC only. All surveyed private clinics (7/7) provided CEmOC, while 57% (4/7) of surveyed public facilities provided CEmOC. The four public facilities offering CEmOC included Rangpur Medical College Hospital, which provided all CEmOC services, the Maternal and Child Welfare Clinics of Rangpur and Gaibandha, which provided CEmOC minus assisted vaginal delivery, and Gaibandha District Hospital, which offered CEmOC minus assisted vaginal delivery and removal of retained products. The three surveyed private clinics in Rangpur offered all CEmOC services. Among private clinics in Gaibandha, three provided CEmOC minus assisted vaginal delivery, while the fourth offered CEmOC minus assisted vaginal delivery and removal of retained products. The three sub-district hospitals provided BEmOC only, with the sub-district facility in Rangpur providing six basic functions (minus assisted vaginal delivery) and the two sub-district facilities in Gaibandha providing five basic functions (minus removal of retained products and assisted vaginal delivery).

**Figure 2:**
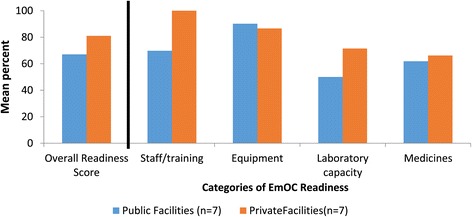
**Facility readiness for emergency obstetric care provision among 14 surveyed health facilities.** Figure 2 illustrates facility readiness scores for EmOC, overall and among the four categories, for the 14 surveyed health facilities in Gaibandha and Rangpur Districts between October 2011 and January 2012, by sector.

Figure 2 shows the average EmOC readiness score for surveyed facilities in each sector (public or private). Overall, the seven surveyed facilities in the private sector exhibited higher EmOC readiness (81%; range: 63%-91%) compared to the public sector (67%; range: 48%-91%) (p = 0.040). Among the readiness categories, surveyed private clinics scored higher in staffing and laboratory capacity for blood transfusions (100%; 71%) compared to public facilities (69%; 50%) (p = 0.036; p = 0.032). For the categories of equipment and medicines, public facilities had similar average scores (90%; 66%) compared to private clinics (86%; 62%) (p = 0.072; p = 0.061). The following sections describe and compare EmOC readiness in surveyed public to private facilities. These results are from surveys that included all seven public facilities located in the two districts and the seven most frequently visited private facilities (representing 83% of the visits made to private clinics by study participants for reported pregnancy-related complications).

### Staffing

**Figure 3:**
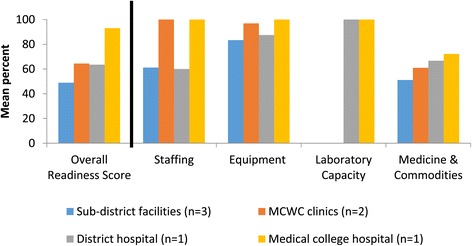
**Facility readiness to provide emergency obstetric care among 7 surveyed public facilities.** Figure 3 illustrates facility readiness scores for EmOC, overall and among the four categories, for the 7 surveyed public facilities in Gaibandha and Rangpur Districts between October 2011 and January 2012, by type of facility.

Among surveyed public facilities, overall EmOC readiness in staffing ranged from 60% in sub-district facilities (n = 3) to 100% in the medical college hospital (n = 1) (Figure 3). Staffing requirements for CEmOC are based on 24-hour coverage of a surgeon and anesthesiologist for obstetric surgery (Table 3). At the sub-district facilities and district hospital, 60% of the physician positions (senior or junior consultants) remained vacant. There was no surgeon at the district hospital, but rather a resident medical officer trained in provision of obstetric surgery. Surgeries were provided two days a week only to accommodate the availability of the part-time anesthesiologist, whose permanent position was with a larger hospital located 71 km southwest of Gaibandha. Even though the district hospital was considered to have CEmOC availability as defined by the provision of signal functions over the past three months, these surveys revealed that the hospital did not have full readiness for CEmOC provision due to limitations in staffing. In six of the surveyed public facilities, doctors were scheduled on-duty at public facilities during normal hours (8 AM to 2:30 PM Saturdays through Thursdays) and were on-call after hours for private clinics outside of government hours.

Because the seven surveyed private clinics had at least one anesthesiologist or surgeon on staff for 24-hour coverage (Table 4), average staffing scores were higher in private facilities (100%) compared to public facilities (69%) (p = 0.036) (Figure 2). In private clinics, staffing allocations were determined by individual managing directors rather than by the central health ministry. Three of the seven surveyed private clinics were started by husband-wife medically-trained couples, while the remaining four clinics we surveyed were started by associations of doctors. While all nurses working in public hospitals were required to have a degree in nursing, many private clinics also hired nurses with no degree in nursing but who were given on-the-job training (called “non-diploma” nurses).

### Equipment and supplies

In the equipment and supplies category, the average score for the public sector (90%) was similar to the average score for the seven surveyed private facilities (86%) (p = 0.072) (Figure 2). Among all public facilities (n = 7), equipment scores were above 80% (Figure 3). Assessments indicated that all three sub-district facilities had the functional equipment necessary for obstetric surgeries, including fully stocked operation theaters backed up with generators. Administrators at public facilities in Gaibandha (n = 5) explained that donors such as UNICEF and AusAid had helped to increase the availability of equipment required for anesthesia, surgery, and newborn resuscitation. Despite availability of required equipment and supplies, the sub-district facilities were unable to perform obstetric surgeries due to lack of full-time anesthesiologists and surgeons.

While six of seven public facilities had at least one functioning ambulance on site, only three out of the seven private clinics had an ambulance on-site. For all of the surveyed facilities, private ambulances were typically stationed close by and were available to patients for a fee. The average cost of transport from public facilities in the town of Gaibandha (n = 2) to the medical college hospital in Rangpur was 1,600 Taka (US$20) using the facility ambulance, compared to 1,500 Taka (US$18) using private ambulances. Discounts were offered for the poorest clients (discussed later).

### Medicines and commodities

**Figure 4:**
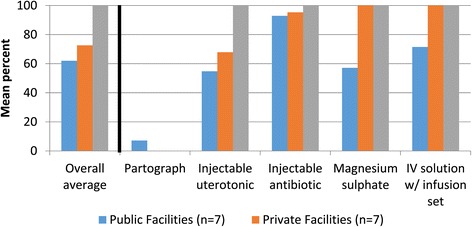
**Coverage of EmOC-specific medicines and commodities in 14 health facilities and 28 pharmacies surveyed.** For health facilities and pharmacies of Gaibandha and Rangpur Districts surveyed between October 2011 and January 2012, Figure 4 illustrates coverage of EmOC-specific medicines and commodities.

Figure 4 shows the coverage of medicines and commodities required for EmOC by sector, as well as in adjacent pharmacy shops. Public facilities reported providing medicines for free if in stock, while medicines at private sector clinics and pharmacies required purchase. Coverage was measured by the presence of medicines in facilities or pharmacies on the day of the visit. Average coverage of EmOC-specific medicines and commodities was 100% in surveyed pharmacies, 66% in the seven surveyed private clinics, and 62% in surveyed public facilities (p = 0.034). Pharmacies scored 100% in every category, meaning that the seven surveyed pharmacies (n = 28) had all medicines required for EmOC on the day of the visit. However, only two-thirds of the surveyed pharmacies had refrigerators with back-up generators needed for constant refrigeration of drugs such as oxytocin. Pharmacies, which do not routinely carry supplies for labor monitoring, were not scored for supply of partographs.

Surveyed private clinics (n = 7) scored higher than the public facilities (n = 7) on availability of magnesium sulfate (100% in private clinics and 57% in public facilities) and IV solution with infusion set (100% in private clinics and 71% in public facilities). Surveyed private and public facilities had similar scores on availability of injectable uteronics (58% in private clinics and 56% in public facilities) and injectable antibiotics (95% in private clinics and 92% in public facilities) (Figure 4). Coverage of the partograph was low in both sectors (7% in public facilities and 0% in private clinics).

The category of medicines and commodities also included the provision of blood from blood banks or from other sources, as well as provision of required screening tests [5]. In the study area, there was no centralized blood bank; only the tertiary medical college hospital maintained a facility blood bank. In the other 13 facilities, clinicians required families to procure blood before surgeries could be performed. For blood acquisition, patients could either exchange blood through voluntary associations or purchase blood from unregulated private diagnostic centers. Of the annual volume of blood that was procured in Gaibandha in 2010 (1,700 bags), voluntary exchange programs provided 66% of this total, while private blood banks provided 34% [24].

The largest voluntary exchange association in Bangladesh is called *Sandhani*; through this group families can exchange donated blood for the blood type needed for surgery. *Sandhani* attendants perform the required screening tests (except for malaria screening) and blood typing at their offices for a cost of 250 Taka (US$3.05). For patients at the district hospital, blood bags were provided for free and malaria screening was performed. Patients at other facilities had to purchase blood bags at a cost ranging from 120 to 170 Taka (US$1.46-$2.07) per bag.

Patients could also choose to purchase blood from private diagnostic centers, although these centers were not authorized to store blood and paid individuals to provide blood for patients. These private centers did not provide screening for transfusion-transmissible infections except for Hepatitis B. The Civil Surgeon, who serves as the head of public-sector health services in the district, noted the difficulty in enforcing regulations and standards among private diagnostic centers.

### Laboratory capacity

For CEmOC services, laboratory capacity was defined based on the provision of cross-matching tests, which determine the compatibility between donor and recipient blood prior to blood transfusion. While the medical college hospital and the district hospital in our sample (n = 2) offered cross-matching, none of the other public facilities (n = 5) offered this service (Figure 3). The seven surveyed private clinics in Gaibanda (n = 4) offered cross-matching. Owners of the three surveyed private clinics in Rangpur (n = 3) did not offer cross-matching at their health facilities. If cross-matching was not available at a health facility, patients could pay for this service performed at nearby private diagnostic centers.

### Access to care: costs of services

The sector in which services are provided is known to affect the costs borne by the patient as well as the compensation of the provider [25]. We chose to compare the cost of C-section among facilities offering CEmOC services. Table 5 shows the costs of registration fees and C-sections at CEmOC facilities (n = 11), excluding the costs of medicines or supplies. While two of the four public facilities that offered CEmOC charged nominal registration fees (US$0.06 at the district hospital and US$0.18 at the medical college hospital), the cost of the C-section procedure was reportedly free at all four public facilities. As of March 2011, the Gaibandha Private Clinic and Diagnostic Center Owners’ Association had standardized the costs of C-section among private clinics in Gaibandha to be 5,500 Taka (US$67), which were posted in placards in the lobbies of all private clinics in Gaibandha. For the three private clinics surveyed in Rangpur, the fees for C-section ranged from 6,000 Taka to 8,500 Taka (US$73.17-US$103.66).

**Table 5:** **Costs of Caesarian-section procedure and registration fees at 11 surveyed health facilities providing comprehensive emergency obstetric care**

**Type of clinic**	**Cost of C-section (Taka)**	**Cost - US $**
Sub-district hospitals (n = 3)	N/A	N/A
Maternal and Child Welfare Clinic, Gaibandha	Free	-
Maternal and Child Welfare Clinic, Rangpur	Free	-
District Hospital, Gaibandha	5	$0.06
Medical College Hospital, Rangpur	15	$0.18
Private Clinic, Gaibandha	5500	$67.07
Private Clinic, Gaibandha	5500	$67.07
Private Clinic, Gaibandha	5500	$67.07
Private Clinic, Gaibandha	5500	$67.07
Private Clinic, Rangpur	6000	$73.17
Private Clinic, Rangpur	8000	$97.56
Private Clinic, Rangpur	8500	$103.66

Table 5 represents the costs of Caesarian section procedures at the 11 surveyed CEmOC facilities in Gaibandha and Rangpur Districts between October 2011 and January 2012. Costs were reported by health administrators and did not include the costs of medicines or supplies for the C-section procedure. Estimated costs are based on an exchange rate of 82 Taka/$1 USD (rate as of January 20, 2015 from Bangladesh Bank [26]).

These estimates did not include the costs of medicines and supplies. At public facilities, medicines were reportedly free, though facilities were not always stocked in essential EmOC medicines (shown in Figure 4). All medicines required for C-section were found to be available at nearby pharmacy shops (n = 28), where costs ranged from 2000 to 2500 Taka (US$24.39-US$30.49).

Managing directors of private clinics explained that the poorest women were given a 70% discount on costs of C-section services, including the cost of the procedure, transport for referral, medicines, supplies, and laboratory tests. These women were identified through their membership with BRAC, an NGO dedicated to poverty alleviation through programs in microcredit, health, and other services to women [27]. Membership cards were assigned to women according to their socioeconomic group: rich, medium poor, and hard-core poor. To help manage this process, two BRAC program organizers worked in the district hospital and maternal and child welfare clinic in Gaibandha to identify program beneficiaries and assist in navigating them through the hospital system.

### Constraints to EmOC provision

In the open-ended portion of these assessments, we asked respondents to discuss the main constraints to EmOC provision. Eighty percent of respondents identified staffing constraints in rural areas as the main barrier to EmOC provision. While half of respondents mentioned the need for essential medicines and commodities to be in stock at public facilities, respondents said that constraint was partially met by the ready availability of these medicines at pharmacy shops. Half of the surveyed health facility administrators (n = 7) cited the need for a 24-hour centralized blood bank at the district level from which residents can acquire blood without donation or purchase to decrease the complicated process of safe blood acquisition.

Respondents from public facilities discussed the difficulty in retaining the anesthesiologists and surgeons needed to perform obstetric surgeries. Although specialists were typically allocated to sub-district complexes by the MOHFW, health administrators at these facilities explained that these specialists were difficult to retain. All three head administrators of the sub-district facilities commented that retaining specialized staff in rural health facilities was difficult due to the reduced scope for clientele and private practice and poorer quality of schools and markets compared to urban areas. They explained that doctors were able to change their postings for positions in urban areas through lobbying with networks and doctors’ associations.

To address the acute shortage of surgeons and anesthesiologists in rural areas, some facilities focused on task sharing of these responsibilities to non-specialist doctors and lower-level health cadres. The district hospital in our sample, in fact, had already implemented task-sharing to allow for provision obstetric surgery by a junior doctor with four weeks of training in surgical provision rather than a specialist. However, the MOHFW respondents (n = 2) and the Civil Surgeons (n = 2) cautioned that doctors’ associations had lobbied against task sharing of their responsibilities to lower-level cadres due to concerns over compromised quality of care. The policymakers stated that such opposition may hinder the ability to implement this policy at the national level.

## Discussion

Among the study population in Gaibandha and Rangpur Districts, this analysis provided information on the availability and readiness for emergency obstetric care at the most commonly visited health facilities for pregnancy-related complications. Readiness for EmOC provision in the public sector appeared to be limited primarily by staffing constraints, particularly the retention of specialists in rural areas. Despite the lower availability and readiness for EmOC provision compared to private clinics, public facilities appeared to be more accessible to clients in terms of costs of services and geographic location. Interviews revealed suggestions for improvement of EmOC readiness through strategies for recruitment and retention at public facilities, which remain more accessible to women, especially those in the lowest wealth quintiles.

Staffing constraints remained the major barrier to provision of services in the public sector, as described by facility assessments in sub-districts of Bangladesh such as Khulna, Sylhet, and Habiganj [9,28]. The 2012 *Health Bulletin* indicated that 50% of all senior consultant positions nationwide were vacant, while 58% of the junior consultant positions remained vacant [8]. Other studies have described the acute crisis in manpower in the public health sector, particularly of surgeons and anesthesiologists [9,10]. Even when rural facilities were fully equipped to provide obstetric surgery, they were unable to perform this service due to lack of specialized staff.

While Anwar et al. have suggested the implementation of non-financial incentives (e.g. increased promotion opportunities for doctors working in rural areas) to increase retention of skilled staff in rural areas [9,10], policymakers have cautioned that such changes would have to be authorized through numerous levels of government systems as well as doctors’ associations. Due to the centralized system of health sector programs in Bangladesh, policies that impact staffing levels at sub-district and district levels are determined nationally by the Ministry of Health and Family Welfare’s Directorate General of Health Services [10]. The MOHFW also determines staffing allocation among public based on the number of beds per facility rather the volume of deliveries performed at facilities. Although the two Maternal and Child Welfare Clinics in our sample accounted for more than a third of all deliveries in surveyed public facilities, these clinics were allocated only two doctors. Health administrators at the two MCWCs explained that the misallocation of doctors coupled with the high volume of deliveries created extremely crowded patient conditions.

Although our surveys indicate that medicines essential for EmOC are available for purchase at pharmacies, medicine availability in public facilities remains important for the poorest clients. Administrators at public facilities explained that they periodically experienced stockouts of uterotonic drugs (oxytocin, misoprostol) and magnesium sulfate though all are listed as essential drugs for Bangladesh, are included in standard treatment guidelines, and are manufactured in country [29]. In a survey administered to MOHFW officials, the Maternal and Child Health Program (MCHIP) reported that only 55% of district facilities and 38% of sub-district complexes had oxytocin in stock, while 19% of public facilities were reported to have magnesium sulfate in stock [29,30].

In this sample, all of the facilities offering comprehensive emergency obstetric care were located in the main towns. For a population of 2.3 million, the concentration of CEmOC facilities in the main towns necessitated travel up to two hours to reach surgical care. Women who sought surgical EmOC services from the more financially accessible public facilities in Gaibandha had two options: a district hospital that offered surgery only two days a week, or a Maternal and Child Welfare Clinic with the highest volume of deliveries of any facility in Gaibandha. If these facilities were full or otherwise could not provide surgery, patients could choose to visit the tertiary medical college hospital in Rangpur Town, located another two hours away by motorized transport, or to a private clinic, where the cost of C-section was up to more than 100 times higher than in the public sector. The difference in the cost of C-section in the private sector is likely to be prohibitive for women in the lowest wealth quartiles. Studies show that wealth inequities in maternal care utilization remain strong, as women of lower SES are less likely to access every maternal health care service, including antenatal care, institutional delivery, and skilled attendance at delivery [31-33]. The distance to CEmOC facilities and cost of services, coupled with the lack of specialized staff in public facilities, may compound delays to reaching and receiving care.

### Limitations

These surveys were focused on availability and readiness for EmOC provision among a selection of health facilities in Gaibandha and Rangpur Districts. While surveys included all public hospitals in the two districts, we purposively selected the most frequently visited private clinics to characterize EmOC readiness at high-volume health facilities. Recognizing that facility utilization is influenced by quality and level of care provided [3,9], these selected private facilities may have higher service quality compared to the private clinics that were not surveyed in this study. While the surveyed private clinics may not be representative of all private clinics, they represent the high-volume health facilities that capture the majority of women who seek private facilities for obstetric complications. For districts with limited health budgets, focusing on service improvement at this selection of private clinics is likely to reach a large proportion of women of reproductive age with pregnancy-related complications.

These findings resonate with surveys in Khulna, Sylhet, and Habiganj Districts, which found lower levels of EmOC availability in public facilities compared to national reports and described staffing constraints as the main barrier to EmOC provision [9,10,28]. The surveys presented in this paper focused on availability and readiness to provide EmOC and did not assess quality of care or quality of medicines. Since the locations of informal health providers were not recorded in the GIS database, we were unable to assess EmOC provision among informal providers in this analysis. However, the focus on EmOC availability at health facilities is in accordance with current WHO recommendations [5,15,16]. These cross-sectional assessments may not have captured changes in service availability over time. While we presented costs of services, analysis of clients’ ability to pay was beyond the scope of this analysis.

### Recommendations

For equitable provision of EmOC in these rural districts, strategies to improve EmOC readiness at public facilities is needed. We present recommendations that may improve EmOC availability and readiness among public facilities in Gaibandha and Rangpur. Because changes must be approved nationally due to the centralized system of health provision in Bangladesh, recommendations are given at the national level.

1. In the public sector, incentives should be established to recruit and retain specialists, particularly surgeons and anesthesiologists, in rural areas.
  Because Bangladesh has a centralized health system, decisions on allocation of positions for specialists are determined by the Ministry of Health and Family Welfare [10]. Given the difficulty in establishing financial incentives for one particular government cadre, the MOHFW may consider focusing on non-financial incentives, such as advancements for career promotions and trainings, to improve retention among doctors serving in rural areas. While research is ongoing on the impact of non-financial incentives on staff retention in resource-limited settings [34], data from programs in rural districts of Sri Lanka and Thailand suggested that non-financial incentives including career development, training opportunities, and fellowships contributed to retention of specialists in rural areas compared to control areas [35,36]. Moreover, staffing allocations should reflect the real volume of deliveries experienced by facilities rather than solely on the number of beds in the facility. The retention of specialized staff in rural areas, particularly sub-district complexes, could improve geographic accessibility to EmOC in rural areas.
2. The establishment of a centralized blood bank at the district level may decrease the complicated process patients must maneuver to acquire safe blood for operations.

To acquire blood for transfusions, patients and their families must traverse numerous systems and visit multiple clinics. While the MOHFW had increased the number of centers that provide screening tests for blood transfusions, the number of regulated facilities offering storage and acquisition of safe blood for transfusions remained low [37]. The establishment of a 24-hour centralized blood bank at the district level from which residents can acquire blood without donation or purchase may decrease the time required to screen and acquire blood during obstetric surgeries. Such blood banks should be monitored and held accountable to quality standards and practices such as blood screening. Requiring and equipping all CEmOC facilities in both the public and private sectors to provide cross-matching tests may also decrease the time needed for patients to obtain safe blood transfusions.

## Conclusions

Future research may focus on understanding how to improve supply chain management in the public sector for essential medicines. Innovations such as the use of mobile phones to track and replenish essential commodities may be useful [38]. Research on the blood requirements at tertiary, district, and sub-district facilities could assist with planning of centralized blood transfusion and blood collection centers. At the policy level, research is needed on the impact of various non-financial incentive schemes on retention of specialists in rural areas as well as effective context-specific strategies for implementation. Given the growing proportion of institutional deliveries in Bangladesh occurring in the private sector, more thorough, nationally representative surveys of private facilities may be needed to characterize and improve EmOC readiness and provision in this sector.

Recognizing that socioeconomic differentials in care seeking remain important, strategies to improve EmOC availability should focus on retaining staff in the more accessible public sector. The MOHFW’s goal of increasing availability of emergency obstetric care may be difficult to achieve without policies that address the chronic shortages in specialized staff in rural areas. Addressing key areas of improvement such as retention of human resources in the public sector and centralized safe blood acquisition may increase provision of EmOC and equitable access to care for women experiencing obstetric complications in rural, remote settings. Increased EmOC availability and readiness across health facilities may help to reduce additional unnecessary delays in seeking and receiving medical services necessary to treat obstetric complications and, in the most serious cases, prevent maternal death.
